# Revisiting Male Fertility in Livestock: The Case of Bull Sperm RNA

**DOI:** 10.3390/biology14080969

**Published:** 2025-08-01

**Authors:** Rene A. Ramírez-Sosa, Francisco J. Jahuey-Martínez, Monserrath Felix-Portillo, José A. Martínez-Quintana

**Affiliations:** Facultad de Zootecnia y Ecología, Universidad Autónoma de Chihuahua, Chihuahua 31000, Mexico; p363951@uach.mx (R.A.R.-S.); fjahuey@uach.mx (F.J.J.-M.); monserrath.felix@uach.mx (M.F.-P.)

**Keywords:** male fertility, sperm RNAs, pregnancy loss, epigenetics of livestock reproduction, *LYRM4* gene

## Abstract

To achieve the goals of productivity and sustainability across diverse livestock systems, reproductive factors play a pivotal role. Historically, reproductive research has primarily focused on females, as they are responsible for maintaining pregnancy and delivering offspring following oocyte fertilization. However, since the early 2000s, the biological significance of sperm RNAs has been increasingly recognized in various livestock species. These RNAs contribute both genetically and epigenetically at the time of fertilization and during early embryonic development. Multiple types of sperm RNA have been identified in bovine, porcine, ovine, buffalo, and caprine spermatozoa. Notably, transcriptomic profiling has shown potential to differentiate between high- and low-fertility males, even when conventional semen quality values appear normal in both groups. This opens the possibility for more accurate identification of highly fertile sires. Nevertheless, a definitive marker or set of markers has yet to be established, likely due to the transcriptome’s sensitivity to environmental conditions and to the variability in evaluation methodologies. Therefore, global scientific efforts should aim to establish standardized, robust protocols, as sperm RNA represents a promising avenue for enhancing the sustainability of animal production systems.

## Simple Summary

Improving how we breed livestock is essential for both food production and protecting natural resources. While most research has focused on female animals, scientists are now discovering that sperm also plays an important role beyond simply carrying DNA. In particular, molecules called RNA found in sperm contribute to controlling early embryo development in animals like cows, pigs, sheep, and goats. Studies have shown that the types and amounts of RNA in sperm may help tell which males are more likely to produce healthy offspring—even when their sperm looks indistinguishable under a microscope. This could help producers choose the best animals for breeding. However, researchers have not yet found a clear test to carry this out reliably, partly because RNA can change depending on the environment or how it is measured. To make progress, scientists around the world need to agree on the best ways to study sperm RNA. If successful, this research could lead to the development of better tools for selecting top-quality breeding animals, which would support more sustainable and efficient livestock farming.

## 1. Introduction

One of the main challenges in livestock production systems is the improvement of reproduction efficiency. To address this challenge, many countries worldwide have increasingly introduced the use of different assisted reproductive technologies (ARTS). However, there is still a wide margin for progress in this regard. According to data published in 2024 by the Data Retrieval Committee (DRC) of the International Embryo Technology Society (IETS), around 2.4 million embryos were collected or produced from farm animals in 2023, representing a global increase of 13.2% compared to 2022. In North American beef cattle, 2,151,039 oocytes were obtained by ovum pick-up (OPU), of which 26.41% (568,164) generated transferable embryos. A total of 550,052 embryos were subsequently transferred, either fresh or frozen [1]. According to studies conducted over the past 25 years, in the USA, only 27% of cattle that receive in vitro-produced embryos give birth to live offspring, with approximately 60% of these pregnancies lost within the first six weeks of gestation [2]. As for the evaluation of artificial insemination, data show that of 10,466,000 cows inseminated with frozen semen in the USA, a fertility rate of 68% was achieved, whereas in Mexico, from 900,000 artificially inseminated cows, the fertility rate was 40%, based on a 59-day non-return to estrus [3]. These findings highlight a significant opportunity for the development of technologies aimed at both increasing fertility and reducing embryonic losses in animal production systems. In the last two decades, different research works have demonstrated that failures in fertility and pregnancy maintenance are due to the male [4,5,6,7].

In addition to providing half of the genetic material to the zygote at the time of fertilization, the mammalian sperm also delivers different classes of RNA, many of which are related to fertility in a broad sense [8]. Therefore, it has been proposed that the analysis of transcript types and their concentration levels in the sperm of individuals divergent in fertility could generate knowledge to increase the accuracy in the selection of reproductive males in livestock. Thus, this review aims to (1) critically analyze how fertility failures—particularly embryonic losses—significantly impact the sustainability of livestock production systems; (2) examine the role of the male in these losses; and (3) explore the potential of sperm transcriptomic studies to more accurately assess male fertility.

## 2. Importance of Reproductive Efficiency

Reproductive efficiency is crucial for the sustainability of livestock production. Several studies have shown that various factors related to cattle reproduction significantly affect the economic success of production systems [9,10,11]. It has been reported that, in the United States alone, reproductive losses in beef cattle can amount to as much as USD 2.8 billion annually [12]. Days open, conception rate, number of services per conception, and age at first calving are key variables for accurately assessing reproductive success [13].

Reproductive failures can occur at any stage of the reproductive process, even before insemination. A live calf is only born when all phases, from fertilization to parturition, proceed successfully, thus maximizing the conception-to-loss ratio to improve reproductive efficiency [14].

One way of supporting the sustainability of livestock production that has garnered significant attention is to reduce embryonic loss [15]. Embryonic loss can occur at any time during gestation, although the highest incidence is within the first 30 days. A study estimated that the economic cost of a single pregnancy loss in an intensive dairy production system exceeds USD 250 if it occurs between 45 and 90 days of pregnancy, with costs increasing as gestation progresses [16].

Fertilization rates generally exceed 90% in heifers and cows with moderate productivity, goats, and camelids. However, in high-producing dairy cows, rates are slightly lower (85–89%) due to their higher metabolic demands [17]. Despite successful fertilization, embryonic losses may reduce pregnancy rates to under 50% in both beef and dairy cattle. Causes of pregnancy loss include pre-existing conditions and genetic abnormalities, as well as external factors such as stress, inadequate nutrition, and disease [18]. Importantly, poor gamete quality—particularly subfertile semen—or improper handling can also originate such losses [18].

Although worldwide research advances in reproductive biology have helped identify factors that contribute to reproductive failure, embryonic mortality, and pregnancy loss, accurately determining the cause and timing of embryonic death—especially early in gestation—remains challenging. In general, in dairy cattle, approximately 20% of pregnancy losses occur between 19 and 27 d post insemination [19], while in beef cattle around 15% occur between 16 and 32 d [15].

Most of the research in the field of bovine reproductive physiology has focused on understanding and improving cow fertility [20], primarily on the resumption of cyclicity after calving, increasing pregnancy rate through artificial insemination and calving intervals. Comparatively, male fertility has received less attention [21]. However, the subfertility of bulls used in artificial insemination or in vitro fertilization programs negatively impacts the overall reproductive performance of the herd, even though it is difficult to determine precisely how much of a herd’s low reproductive efficiency can be attributed to the male. Nonetheless, the fertility of a single bull can influence reproductive outcomes across multiple countries. Therefore, there is a pressing need to identify paternal biomarkers that can accurately reflect the male’s contribution to early embryonic development, with the goal of improving overall reproductive efficiency [20].

## 3. Male Contribution to Embryonic Loss

Historically, sperm was viewed merely as a vehicle for half of the genetic material. It is now known that sperm contributes to both genetics and epigenetics at the time of fertilization, not only through DNA but also through important molecular processes such as DNA damage repair, DNA methylation, or determinant molecules such as protein markers, plasma seminal components, and specific RNAs with biological functions [22,23]. During spermiogenesis, several mRNAs are produced and sequestered in ribonucleoprotein complexes before transcriptional quiescence [24]. The sperm cytoplasm is remarkably small and lacks ribosomes, which impedes translation into proteins. Furthermore, the chromatin is highly condensed due to the replacement of histones with protamines which facilitate the dense organization of genetic material, thus limiting transcription [22]. Sperm carry diverse RNAs, including mRNA fragments and regulatory RNAs such as siRNA (small interfering RNAs), miRNA (microRNAs), piRNA (Piwi-interacting RNAs), and lncRNA (long non-coding RNAs), which contribute epigenetic signals that influence embryonic development and regulate gene expression in the new organism [25].

Franco et al. [26] have shown that even semen deemed normal via conventional analysis (motility, morphology, etc.) can vary widely in fertility outcomes like pregnancy establishment and loosing rate. For example, bulls used in artificial insemination (AI) may show large variation in pregnancy establishment and loss rates. Although factors intrinsic to the female and external factors such as farm location were considered, the authors classified the bulls according to early embryonic mortality (defined as losses occurring between 24 and 31 days post AI) and late embryonic mortality (losses between 31 and 60 days post AI), indicating that visual semen quality assessment is insufficient. Identifying transcriptomic markers could help predict which bulls are more likely to sustain pregnancies and select those that increase reproductive efficiency. In another study [5], sires with differing in vitro blastocyst production rates were classified as high- or low-performing. Since a major developmental arrest occurred at the five- to six-cell stage, embryos at this stage were analyzed. The results showed that embryos derived from low-performing sires exhibited increased autophagic activity. The study highlights the male contribution to early embryonic development during the first week of gestation, as transcript levels in four-cell embryos varied between groups. Embryos from high-performing sires displayed elevated levels of mRNAs involved in transcription, chromosome segregation, and cell division, whereas embryos from low-performing sires showed altered expression of transcripts critical for proper embryonic development.

When breeding males are examined for reproductive fitness before each breeding season, it should be noted that even if a male was classified as adequate the previous year, there is no guarantee that it will achieve the same status the following year [27]. The use of AI and other ARTS requires accurate assessment of semen quality and fertilization effectiveness, making these aspects a matter under constant review. Several tests have been developed to predict semen quality, but no single, highly reliable test is currently available. In this sense, sperm transcriptomic profiling has gained importance among animal reproduction research specialist, providing ever-growing evidence of the involvement of sperm RNAs not only in spermatogenesis and sperm function but also in the events following fertilization such as oocyte function, embryogenesis, trophectoderm development and pregnancy establishment [4,28]. Therefore, male selection should be more comprehensive and evaluation of male fertility through transcriptomic analysis could provide a more sensitive and sophisticated technological tool [27], which, combined with traditional analysis of semen quality, such as motility and morphology, and Computer-Assisted Sperm Analysis (CASA) traits, would allow a broader and more complete view of a male’s reproductive potential.

## 4. Reported Sperm-Borne RNA Types

Once the notion that RNAs present in sperm were merely remnants of spermatogenesis was eradicated, an active wave of research began focusing on the characterization of various types of sperm-borne RNAs in different livestock species, and their potential roles in male fertility and successful pregnancy outcomes [8,29,30,31,32,33,34,35,36,37,38]. The RNA content of spermatozoa includes both messenger RNAs (mRNAs), also known as coding RNAs, and non-coding RNAs (ncRNAs). Non-coding RNAs are typically classified into long non-coding RNAs (lncRNAs; >200 nucleotides) and small non-coding RNAs (sncRNAs; <200 nucleotides). The latter group includes various subclasses including miRNAs, piRNAs, transfer RNAs (tRNAs), tRNA-derived fragments (tRFs), ribosomal RNAs (rRNAs), endogenous small interfering RNAs (endo-siRNAs), small nucleolar RNAs (snoRNAs), small nuclear RNAs (snRNAs), and NF90-associated snRNAs. Additionally, circular RNAs (circRNAs) have also been identified in sperm. snRNAs mediate pre-mRNA splicing, whereas snoRNAs guide chemical modifications of rRNAs and tRNAs, and some snoRNAs also have gene-silencing roles. piRNAs protect germline genome integrity by silencing transposable elements. siRNAs, derived from double-stranded RNA, mediate mRNA degradation and contribute to antiviral defense and genome stability. miRNAs fine-tune gene expression by repressing translation or degrading target mRNAs; they are essential in development and disease, acting through partial complementarity with mRNA 3′ UTRs. lncRNAs regulate gene expression through chromatin remodeling, transcriptional control, nuclear organization, and post-transcriptional mechanisms. Some of them function as miRNA sponges or even encode small peptides that switch gene transcription on or off. circRNAs regulate gene expression acting as miRNA sponges, while tRFs affect transcript stability [39]. Functional annotation and ontology analyses indicate that sperm-borne RNAs are involved in a wide range of metabolic and regulatory pathways, which are summarized in Table 1.

Epigenetic Functions (Histone Modification, Chromatin Organization), Embryonic Development.

In cattle, protein-coding RNAs dominate the sperm transcriptome. According to Selvaraju et al. [40], 95.49% of the functionally annotated transcripts are protein-coding, while 3.23% correspond to mitochondrial rRNAs (Mt_rRNA). The remaining 1.28% include other RNA types such as misc_RNAs (0.55%), pseudogenes (0.29%), miRNAs (0.19%), rRNAs (0.16%), processed pseudogenes (0.05%), snRNAs (0.04%), and snoRNAs (0.01%). A more detailed analysis of the sncRNA population revealed that the most abundant biotypes are piRNAs (26%), rRNA fragments (25%), miRNAs (20%), and tRFs (14%), with minor contributions from mRNA fragments (0.4%), unclassified RNAs (6%), and other sncRNAs (8%) [29]. A transcriptome-wide study in crossbred bulls [41] confirmed that protein-coding RNAs predominate, while non-coding RNAs such as rRNAs, miRNAs, and misc_RNAs rank among the most abundant transcripts, although in lower proportions.

In sheep [37], small RNA profiling in rams with high fertility (HF) and low fertility (LF) revealed distinct distributions. In LF males, miRNAs account for 7.12% of reads, repetitive element reads 17.3%, exonic reads 18.04%, and intronic reads 13.99% (5.74% positive strand, 8.26% negative). In HF rams, miRNAs represent a lower proportion (3.78%), while repetitive elements and exonic/intronic reads are comparable (17.54%, 17.02%, and 15.63%, respectively). The remaining reads fall into a broad “other” category (24.64% in LF, 27.93% in HF).

## 5. Sperm Transcriptomic Profiling and Its Potential to Diagnose Male Fertility

Transcriptomic studies investigate the complete set of RNA transcripts existing in one or more cells—the transcriptome—at a given time. This approach has benefited from the latest high-throughput analytical methods such as microarrays, RNA sequencing (RNA-seq), single-cell RNA-seq, spatial transcriptomic sequencing, and degradome sequencing, also known as Parallel Analysis of RNA Ends (PARE) or Genome-wide Mapping of Uncapped Transcripts (GMUCT), among others, allowing for genome-wide profiling of transcripts [55,56,57] beyond just messenger RNAs, thus contributing to the discovery of a massive pool of non-coding RNAs [58]. These technologies, in addition to common techniques, such as the reliable and sensitive Reverse-Transcriptase Quantitative Polymerase Chain Reaction (RT-qPCR) for accurate quantification of RNAs, Stem-Loop PCR for short-length miRNAs and siRNAs, in situ hybridization for spatio-temporal expression patterns, reporter assays or the classical Northern blot for the detection of precursor and mature RNAs, have made it possible to characterize several types of sperm RNA, such as tRNA, rRNA, mRNA, snoRNA, snRNA, sncRNA, lncRNA, and mitochondrial RNA (mtRNA), and to determine which RNAs are found in a particular tissue or group of cells, providing insight into gene expression levels, functions, and regulatory mechanisms [55]. The high-throughput technologies used for RNA analyses generate vast amounts of complex data which, particularly for the discovery of ncRNAs, can be difficult to interpret. In this context, the integration of Machine Learning (ML) with ncRNA research has emerged as a powerful tool for the elucidation of ncRNA-based biomarkers [57,59]. A recent comprehensive review on the biogenesis, discovery, and mechanisms of regulatory ncRNAs is provided by [57].

Sperm RNA has been identified in many mammalian species, including humans, cattle, pigs, buffalo, sheep, horses, and rodents. Some functions of sperm RNA include contributing to fertilization by delivering paternal RNA to the oocyte, participating in mitochondrial translation signaling [60], and playing key roles in the developmental stages of oocyte cell division after fertilization, placental development [40], zygote formation [32], early embryonic development [61], and even transgenerational epigenetic inheritance [62]. Many of these types have already been identified in bovine [8] and porcine sperm [63].

Gene silencing studies have demonstrated that certain sperm RNA transcripts have specific functions in reproductive processes. For example, *Izumo1* is a sperm-specific gene that encodes an essential protein for sperm/oocyte fusion. *Izumo1* knockout mice produce morphologically normal sperm, but these fail to fuse with the oocyte, resulting in male infertility. This gene is essential for the initial stage of zygote formation [64].

Several studies (Table 2) have conducted comprehensive transcriptomic analyses of sperm from different animal species to assess their correlation with fertility. Feugang et al. [65] measured the RNA profiles of sperm cells from 10 Holstein bulls, selected from a pool of 934 based on fertility scores, and grouped into high- and low-fertility categories. Sperm from high-fertility bulls exhibited higher levels of transcripts encoding membrane and extracellular space proteins, while sperm from low-fertility bulls showed reduced levels of transcripts related to transcription and translation factors. Among the most abundant transcripts in spermatozoa were *protamine 1* (*PRM1*) and *casein beta 2* (*CSN2*). Notably, *CD36* (thrombospondin receptor) transcripts were found at significantly lower concentrations in low-fertility bulls, a finding validated through qPCR analysis.

Seven years later, another study on Holstein bull sperm identified 41 differentially expressed transcripts between high- and low-fertility individuals, with expression ratios greater than 10:1 between these groups. Another 574 transcripts had differential expression with ratios greater than 2:1 [44]. One notable distinction between these studies lies in their methodologies, according to what was most available at the time: while Feugang et al. [65] employed microarray analysis, the latter study by Card et al. [44] used RNA-seq, allowing for higher sensitivity and broader transcript detection.

In another study [66], the authors used RNA-seq to compare the transcriptomic profiles of sperm from six Polish Large White boars classified by semen cryopreservation ability, three with good and three with poor semen freezing capacity. The authors reported elevated levels of *FOS*, *NFATC3*, *EAF2*, *BAMBI*, *PTPRU*, *PTPN2*, *ND6*, and *ACADM* transcripts in the sperm of boars with poor cryosurvival, suggesting a molecular signature associated with reduced freezing tolerance. Additionally, a seasonal study in boars analyzed the transcriptomes of sperm collected in winter (n = 5) and summer (n = 5), identifying 14 mRNAs with higher levels and 20 with lower levels in the summer group. Moreover, the authors also report five miRNAs with higher levels and two with lower levels in the summer group [63]. The most abundant miRNA was *miR-34c*, also found in sperm from other mammalian species. This study highlights the significant impact of environmental factors on the regulation of gene expression in sperm and emphasizes the need to account for these variables when the aim is to identify robust biological markers of male fertility.

**Table 2:** Transcriptome analyses in sperm from different species of farm animals divergent for fertility.

Species	Phenotype	Technology	Findings	Reference
Bovine	High vs. Low Fertility	Microarrays	415 of 24,000 transcripts were differentially detected in both groups	[65]
Bovine	High vs. Low Fertility	miRNA sequencing	15 miRNAs were differentially expressed (9 known miRNAs and 6 new ones)	[67]
Bovine	High vs. Low Fertility	miRNA sequencing	85 miRNAs, 2 of them differentially expressed and 9 significantly correlated with the rate of non-return to estrus	[48]
Bovine	High vs. Low sperm motility	Coding and non-coding RNA sequencing	20,875 protein-coding RNAs (19 were differentially expressed); 11,561 were lncRNAs (2517 were differentially expressed)	[68]
Bovine	High vs. Low Fertility	mRNA sequencing	A total of 776 transcripts were found (84 specific to the high-fertility group and 168 specific to the low-fertility group); 176 were at higher levels and 209 at lower levels in the low-fertility group	[69]
Bovine	High vs. Low Fertility	mRNA sequencing	3227 transcripts were found (805 and 5366 unique in the highest- and lowest-fertility group, respectively, and 2944 in common); 41 transcripts had differential levels between groups	[44]
Buffalo	High vs. Low Fertility	Microarrays	51,282 transcripts were found, 113 with higher levels and 596 with lower levels in the low-fertility group	[36]
ovine	Three breeds and high vs. low ejaculate quality	mRNA sequencing	There were different transcripts with differentiated levels between the three breeds and 39 differentiated between ejaculate quality, most of them (33) with lower levels in high-quality ejaculates	[35]
Ovine	High vs. Low Fertility	Small non-coding RNAs sequencing	A total of 1673 known and 627 novel miRNAs were identified, with 227 differentially expressed miRNAs between the HF and LF groups	[37]
Porcine	Fresh vs. Frozen Semen	mRNA and miRNA sequencing	567 mRNAs and 135 miRNAs were differentially expressed	[33]
Porcine	Summer vs. winter ejaculates	mRNA sequencing	14 transcripts with higher levels and 20 with lower levels in the summer group; in addition, 5 miRNAs were down-regulated and 2 were up-regulated in the winter group	[63]
Horse	Fertile vs. Subfertile	Microarrays	437 differentially expressed genes between groups; *OAS1*, *OAS2*, *IL13*, and *IL22RA1* were validated by qPCR, with higher levels in the fertile group	[46]

*miR-34c*, reported as the most abundant miRNA in boar sperm by [63], was also identified by Keles et al. [48] in bull sperm. However, it did not appear among the ten most abundant miRNAs, nor was it differentially expressed between high and low fertility in individuals. Interestingly, *miR-34c* showed a strong negative correlation with the non-return to estrus rate in sex-sorted semen samples and contributed the most to predict this outcome among all miRNAs analyzed in these samples. In contrast, *miR-34c* levels were not correlated with the non-return to estrus rate in conventional non-sorted semen samples. This finding aligns with the previous report by Turri et al. [67] indicating high *miR-34c* expression levels, but there were no significant differences between Holstein bulls with high- and low-fertility levels.

**Figure 1:**
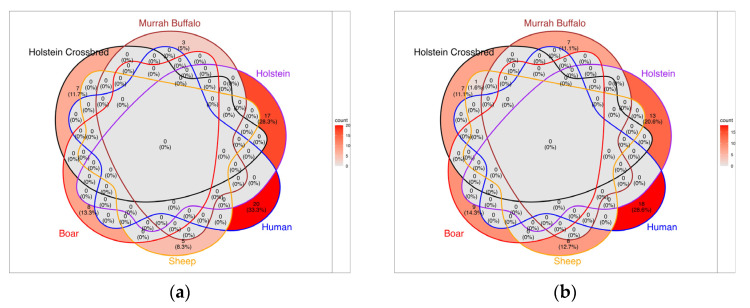
Number of mRNAs that showed differential levels in sperm cells from individuals of different species classified as having low fertility: (**a**) mRNAs with higher levels and (**b**) mRNAs with lower levels.

Several RNA-seq studies analyzing sperm transcriptomes across species have reported lists of mRNAs with higher or lower expression in low-fertility individuals compared to high-fertility counterparts. To identify overlapping transcripts across species, we compiled these lists and mapped each transcript to its Ensembl database ID for cattle, constructing a Venn diagram to visualize the results (Figure 1). Interestingly, no overlap was found among mRNAs reported at higher levels in low-fertility individuals across Holstein bulls [44,65], Holstein crossbred bulls [69], humans [70], sheep [35], boars [63], and buffalo [36] (Figure 1a). Regarding the mRNAs reported to be lower in low-fertility individuals, a single match corresponding to *LYMR4* was consistent in both Holstein crossbred bulls and sheep (Figure 1b). This limited overlap highlights the species-specific nature of sperm transcriptomic profiles and suggests that fertility-associated transcriptomic markers may not be universally conserved.

**Figure 2:**
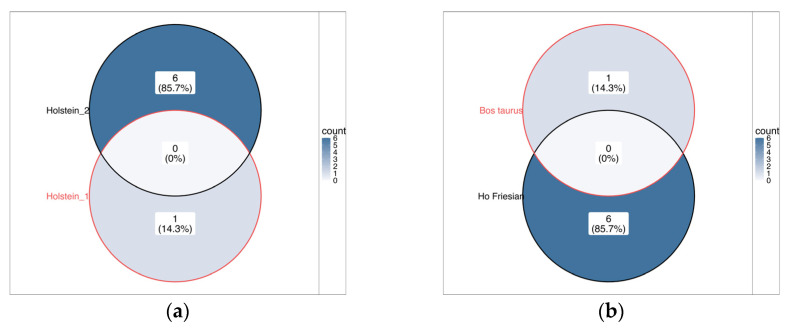
Number of miRNAs with differential levels in sperm cells from individuals classified as having high or low fertility: (**a**) miRNAs with higher levels in individuals with high fertility; (**b**) miRNAs with higher levels in individuals with low fertility.

By performing the same exercise constructing a Venn diagram using the reported lists of miRNAs with higher expression levels in both low- [43,53] and high-fertility individuals [48,51,67,71], we observed that there was no overlap of miRNAs between sperm from Holstein bulls and that from other *Bos taurus taurus* populations (Figure 2). This lack of shared miRNAs across groups further emphasizes the variability of the sperm miRNA profile, even within the same species, and suggests that fertility-associated miRNA markers may be population-specific or influenced by other factors such as genetics, environment, or semen processing methods.

## 6. Characteristics of the *LYRM4* Gene

The *LYRM4* gene, whose mRNA levels were found to be lower in the spermatozoa of both crossbred Holstein bulls and sheep with low fertility, has a length of 92,112 bp structured with three exons divided by two introns. It is located on chromosome 23 in cattle (*Bos taurus*) and encodes a protein of 91 amino acid residues. The LYRM4 protein belongs to the Complex1_LYR-like superfamily, a group of proteins with diverse cellular functions found exclusively in eukaryotes and contain the conserved “LYR” motif near the N-terminus. Also known as ISD11, LYRM4 plays a crucial role in the mitochondrial biogenesis of iron/sulfur (Fe-S) clusters, which are prosthetic groups essential for the function of numerous proteins involved in various biological pathways, including oxidative phosphorylation, the citric acid cycle, iron homeostasis, heme biosynthesis, and DNA repair [72,73]. Given its central role in mitochondrial metabolism, reduced expression for LYRM4 may impair sperm energy production and function and embryonic developmental processes, potentially contributing to reduced fertility.

## 7. Practical Applications of Sperm RNA Biomarkers in Livestock

The study of sperm-borne RNAs has opened a promising avenue for developing more accurate tools to evaluate and enhance male fertility in livestock [4,7,37]. In cattle, transcriptomic and epigenetic profiles of sperm are strongly associated with fertility potential [7,74]. Distinct sets of transcripts and DNA methylation patterns have been related to high- and low-fertility bulls, alongside chromatin remodeling issues and impaired embryo development linked to poor sperm quality [23,74]. Multi-omics studies have revealed substantial differences in thousands of transcripts, proteins, and metabolites, underscoring the molecular complexity behind male reproductive performance [7]. These findings have led to practical applications, particularly in dairy cattle. Sperm RNA and epigenetic markers offer complementary tools to traditional semen evaluation and genetic selection, allowing for early identification of reproductively superior sires and improved diagnosis of subfertility [74]. For instance, DNA methylation markers can be integrated into existing SNP chips for early diagnostic purposes, and epigenetic screening may help predict not only bull fertility but also the fertility potential of female offspring. sncRNAs, such as miRNAs and tsRNAs, show differential expression patterns associated with motility and conception outcomes. Specific miRNAs (e.g., *miR-2385-5p*, *miR-98*) and tsRNAs derived from Gly, His, and Glu tRNAs correlate with both bull and daughter fertility [74]. Moreover, biomarker-guided selection can be applied to screen bulls with superior sperm motility or to optimize in vitro fertilization [74]. In pigs, recognized as translational models, sperm RNA biomarkers such as *EQTN*, *ZP4*, and *SPACA3* have demonstrated high diagnostic accuracy for fertility stratification [4], while sperm-derived circRNAs like circRNA-1572 influence early embryogenesis and zygotic genome activation [75]. In sheep, miRNAs such as oar-miR-200b and oar-let-7d are associated with sperm quality and fertility variation [37]. Despite these promising applications, several limitations must be addressed before widespread implementation. RNA extraction from sperm requires rigorous removal of somatic cells to avoid confounding, and RNA modifications can complicate the detection of sncRNAs. Variability in semen quality, animal age, and ejaculate can affect results, and epigenetic markers identified in one breed or region may not translate across others due to genetic background differences. Moreover, most markers still require large-scale validation and are not yet used in routine practice. Additionally, robust phenotypic recording systems are required [74].

## 8. Conclusions

Different types of RNA contributed by sperm to the zygote play biologically significant roles in early development. Therefore, the discovery of one or more reliable and universal molecular markers in sperm to distinguish fertile from subfertile individuals would have a substantial positive impact on genetic selection and the sustainability of the livestock industry. However, the information compiled in this literature review highlights the complexity and variability in gene expression regulation, underscoring the ongoing need for further research in sperm transcriptomics.

The variability in the RNAs reported across studies may be attributed to multiple factors, including differences in animal populations, experimental methodologies, environmental conditions, or even genetic interactions not yet understood. Thus, incorporating multi-omics approaches—such as transcriptomics, epigenetics, and proteomics—alongside standardized methodologies could yield more conclusive data and facilitate the identification of consistent molecular signatures that serve as robust fertility indicators.

Although *LYRM4* mRNA levels were consistently lower only in crossbred Holstein bulls and sheep with low fertility, the biological functions of its protein product suggest a critical role in embryonic developmental processes. Further investigation of the *LYRM4* gene and its encoded protein in farm animals could provide valuable insights into the male contributions to reproductive efficiency.

## Figures and Tables

**Table 1:** Functional annotation and ontology analyses of the sperm-borne RNAs.

RNA Type ^1^	Before Fecundation	References	After Fecundation	References
mRNA	Translation/Ribosomal Activity, Protein Catabolic Process, Generation of Metabolites, Precursors, and Energy, General Metabolic Pathways, Glycolytic Pathway/Glycolysis and Gluconeogenesis, Pyruvate Metabolism, Citric Acid Cycle, Amino Acid Biosynthesis, Phosphatase Pathway/D-myo-Inositol Phosphate Metabolism, Pyrimidine and Purine Metabolism, AMPK Signaling Pathway, Butanoate Metabolism, Methionine and Cysteine Metabolism, Propanoate Metabolism, Ketone Body Synthesis and Degradation, Nitrogen Metabolism, Oxidative Phosphorylation, Response to Oxidative Stress, EGFR and p38 MAPK Signaling Pathway, Notch and Cadherin Signaling Pathway, Vesicle Trafficking, Ubiquitin-like Protein Ligase Binding, Valine, Leucine, and Isoleucine Degradation, Autophagy and Mitophagy, Proton Transport-Coupled ATP Synthesis, Electron Transport Chain and Aerobic Respiration, Lipid and Glucose Metabolism.	[7,40,41,42,43,44,45,46,47,48]	Ribosome/Translation, Oxidative Phosphorylation, Metabolic Pathways (general), Proton Transport-Coupled ATP Synthesis, Respiratory Chain, Cell Cycle, Proteasome, Spliceosome, Oocyte Meiosis.	[7,27,36,41,49,50]
miRNA	Metabolic Processes (broad category), PI3K/AKT Signaling Pathway, Signaling Pathway Regulating Pluripotency of Embryonic Stem Cells, mTOR Signaling Pathway, Aminoacyl-tRNA Biosynthesis, Vitamin B6 Metabolism.	[45,48,50,51,52,53]	PI3K Signaling Pathway, Blastocyst Metabolism (general), EIF2 Signaling Pathway, Sirtuin Signaling Pathway.	[50]
lncRNA	JAK-STAT Signaling Pathway, Axon Guidance Pathway.	[54]		
circRNA	Epigenetic Functions (Histone Modification, Chromatin Organization), Embryonic Development.	[34]		

^1^ The functional annotation for the non-coding RNA belongs to their target genes.

## Data Availability

Not applicable.
